# Three days inside: exploring reciprocity and trust in a participatory action research community case study in the Netherlands

**DOI:** 10.3389/fpubh.2026.1713934

**Published:** 2026-04-21

**Authors:** Lieke Dalstra, Nikki Jepkema, Amy M. Jeschke, Harold Hofenk, Jonathan Broekhuizen, Fons Van Der Lucht

**Affiliations:** 1 Healthy Ageing, Allied Health Care and Nursing Research Group, Faculty of Health Studies, Hanze University of Applied Sciences, Groningen, Netherlands; 2 Center of Expertise Healthy Ageing, Hanze University of Applied Sciences, Groningen, Netherlands; 3 Urban Issues Research Group, Faculty of Applied Social Sciences and Law, Amsterdam University of Applied Sciences, Amsterdam, Netherlands

**Keywords:** community case study, community involvement, health inequalities, participatory action research, reciprocity, research role, citizen science

## Abstract

Traditional scientific research has produced valuable knowledge, yet its linear, top-down logic often fails to address the complexity of local communities. This results in a gap between knowledge and action, where interventions lack legitimacy and impact. Participatory Action Research (PAR) has emerged as an alternative, bringing residents, professionals, and researchers together in iterative cycles of action and reflection. The promise of PAR lies not only in its methods but in the attitudes and relationships that underpin it. This community case study explores how reciprocity, understood as mutual exchange, trust, and shared responsibility, can be cultivated in PAR. The study focuses on 3 Days Inside, an experiment in the city of Appingedam (Northeast of the Netherlands), a shrinking region marked by demographic decline, earthquakes from gas extraction, and declining trust in institutions. Here, proximity and reciprocity are prerequisites for meaningful participation. Researchers immersed for 3 days, working alongside residents in everyday settings. Participating activities included neighborhood walks, shared meals, and practical collaboration in community spaces. Data were collected on needs and opportunities to improve the healthy living environment through participatory observation, go-along interviews, shadowing, and systematic reflection, with logbooks structured around first-, second-, and third-person perspectives. The case showed PAR is a dynamic process that demands reflexivity, flexibility, and ethical sensitivity. Researchers had to let go of control, acknowledge own positioning and assumptions, and prioritize relationships over outcomes. Gestures of presence and participation proved more powerful than formal conversations. Yet, tensions emerged between relational and transactional practices, between systemic logics and lived realities, and between pursuing research goals and fostering reciprocity. Three days inside highlights that meaningful participation depends less on predefined methods and more on the researcher's attitude—openness, reflexivity, and willingness to share control. The case contributes to broader debates on participation by showing that social impact arises not only from knowledge but from the way knowledge is co-created with residents. For researchers, professionals, and policymakers, the findings underline that genuine participation requires long-term presence, trust-building, and relational ethics. Future research should examine the applicability of this approach in other contexts to strengthen its practical utility.

## Introduction

Traditional scientific research has generated valuable insights, but it often struggles to address complex social realities. Its linear, top-down logic where researchers define problems, design interventions, and measure the outcomes, can fail to meet the specific needs of local communities (1). This contributes to the persistent “knowledge-to-action gap,” in which interventions lack local support and impact remains limited (2, 3), which further limits the effectiveness of such initiatives (1, 4).

Participatory approaches such as participatory action research (PAR) have been developed to bridge this gap by valuing experiential knowledge and involving people from the field without formal research backgrounds in cycles of action and reflection (5). Researchers, professionals, and citizens work together in a cyclical process in which action and reflection alternate. The goal is to achieve concrete changes and improvements in the context of everyday life to complement the laboratory mode of research and the so called “ivory tower of science” (6).

On paper, PAR seems to provide the answer. In practice, however, even PAR can reproduce non-participatory dynamics when carried out within institutional structures that privilege control, efficiency, and standardized methods (7). Researchers may unconsciously reinforce these logics, undermining the very reciprocity they aim to cultivate. This reveals a critical tension: methods alone do not guarantee participation.

Participation is playing an increasingly prominent role in the health and welfare domain in the Netherlands. That is, policymakers and professionals are increasingly recognizing the importance of identifying the wishes and needs of residents at an early stage and actively involving them in both policy development and the implementation of plans (8). This participatory approach is in line with recent government developments in the Netherlands and is explicitly established in legislation and regulations, such as the Environment Act and the Healthy and Active Living Agreement (9, 10). These developments form the broader societal and institutional context in which the case study takes place, and they underscore the urgency of reflecting on how participation can move beyond procedure toward genuine reciprocity.

Yet participation also risks becoming a procedural “box-ticking,” in which mainly the easily accessible residents participate. This can lead to the overburdening of some and the exclusion of harder-to-reach citizens, with the risk of dividing a village, neighborhood or city (11–13). Moreover, questions remain about the extent to which municipalities are genuinely willing to share power and control. How do you ensure that participation is not one-way traffic, but is based on reciprocity and equality? This highlights that participation in itself is not sufficient; what ultimately determines the quality of collaboration is the degree to which reciprocity is realized between researchers, professionals, and citizens.

Reciprocity goes beyond simply involving people in research or decision-making. It entails a mutual exchange in which researchers not only gather knowledge, but also give something back, build trust, and sustain relationships (14). Achieving reciprocity depends less on methodological choices and more on the researcher's attitude (15). Attitude, however, is not a form of knowledge that one can simply read about, understand, and immediately apply; it is tacit knowledge—implicit, embodied, and situated. It is learned not from books, but through experience, practice, and reflection. This raises the key question: how can such an attitude be cultivated in practice?

In PAR, reciprocity is central. It helps balance power relations, foster trust, and ensure that knowledge generates mutual benefits, providing both the ethical foundation for collaboration and the conditions for sustainable outcomes beyond the project (14, 16).

It is precisely within largely non-participatory research infrastructures—shaping the relationships between research, policy, and practice—that the role of the researcher is being redefined. Whereas the traditional researcher acts as an objective observer within a predetermined and often linear (research) design, participatory action researchers act as facilitators, connectors, and reflective partners in a cyclical process of action and reflection (17). Traditional research often takes place in a controlled, defined context and is evaluated on the basis of scientific criteria such as validity, reliability, and generalizability. Research ethics in this context is mainly procedural in nature, laid down in protocols and assessment frameworks (7). In contrast, research ethics in PAR is an ongoing, relational practice of coordination, sensitivity, and care in collaboration (17, 18). These ongoing, relational practices highlight that it is the researcher's attitude—sensitivity, reflexivity, and willingness to share control—that is crucial to embedding genuine participation and reciprocity, rather than relying solely on predesigned methods.

This points to difficult and essential questions: How can attitudes of reciprocity be realized within current research structures? What ethical and relational challenges emerge when researchers step into communities? The aim of this case study is to study the challenges that PAR faces in practice, with a specific focus on community health and social cohesion, where issues of trust, engagement, and collaboration are particularly prominent.

### Three days inside: the case study

These questions and aim are at the heart of 3 days inside, a research experiment designed to explore what happens when scientific work is approached differently: together, in context, and oriented toward change to learn about the challenges that PAR faces in practice. The researchers who are involved are from the community of practice Bridge2Health which aims to learn about the effective implementation of participatory research in the domain of health and wellbeing—under the umbrella term of citizen science (CS)—in the Netherlands (19). Building on this perspective, researchers from Hanze University of Applied Sciences in Groningen and Amsterdam University of Applied Sciences selected the city Appingedam (municipality of Eemsdelta) as their location. This is a shrinking community marked by limited responsiveness, difficulties in engaging residents, distrust of formal institutions, increasing vacancy rates, and declining social cohesion (20). This makes the case study particularly relevant for exploring how participatory research practices—and attitudes of researchers within them—relate to issues of trust between institutions and local communities.

The research experiment consisted of 3 days of participating in the community and learning about the role of participatory action researcher. One of the researchers has been working in this region for more over 5 years as a Professional Doctorate candidate and is doing research on the topic of a healthy living environment and working closely with the local welfare organization and municipality. She was the key figure and ensured that the researchers could easily integrate into this context. The other researchers have varying 2–5 years of experience in PAR in other contexts in the Netherlands connected to Bridge2Health.

During these 3 days, researchers worked alongside residents, building relationships based on trust a shared responsibility. The case raised important questions: What does it mean when control is released? How does the researcher navigate when they no longer determine the direction? What ethical and relational tensions arise? And, most importantly, what can be learned from this about cultivating the attitudes needed for reciprocity in participatory research?

## Context

### Contextual background

The challenges of PAR are particularly evident in shrinking regions such as Appingedam, where demographic and socio-economic developments are putting pressure on quality of life (20). The municipality of Eemsdelta, of which Appingedam is a part, is struggling with population decline, vacancy rates, and a decline in social cohesion (20). At the same time, the region is dealing with the consequences of earthquakes caused by natural gas extraction since 1963. In addition to physical damage to homes, this has led to prolonged uncertainty, stress and deep-rooted distrust of national and local government parties and organizations (21). Broader trends, such as an aging population and increasing pressure on the healthcare chain, also play a role. In regions such as in the Northeast of the Netherlands, the number of older adults people is increasing, while the number of young people is decreasing, which reduces the availability of informal care (20). At the same time, responsibility for health and welfare increasingly lies with municipalities, which requires close cooperation between healthcare and welfare organizations (8). Yet, experience shows that current approaches often rely on a limited group of residents, leading to over-engagement, while traditional nine-to-five working routines prove insufficient for fostering meaningful and sustained community involvement. This is all very urgent in an area where healthy life expectancy is significantly lower than the national average: in 2020, it was 63.6 years in Appingedam, 3.3 years below the national average (22). Perceived good health in 2022 was 61.8% compared to a national average of 69% (23). In a region such as in the Northeast of the Netherlands, where there is widespread mistrust of the authorities, reciprocity in PAR is essential.

### Collaboration and partnership

By not only researching, but also acting together with residents and co-sharing responsibilities, an equal partnership model is fostered that goes beyond one-sided knowledge extraction (17). Paradoxically, the 3 days inside case study shows that letting go of fixed frameworks may ultimately lead to deeper insights through the building of relationships of trust. It is important to recognize that this space for experimentation is partly possible because one of the researchers has been present in this context for more over 5 years, and thus a basic level of relationships was already nurtured beforehand. This long-term involvement has built trust and created space to embed participatory research within existing networks and initiatives.

Describing this community case study brings up tensions. The researchers describe a setting from a certain perspective, while the reality is complex and layered. Finding a balance between academic reflection and doing justice to the relationships built up with organizations and individuals remains an ongoing challenge. How can a researcher analyze critically without damaging existing collaborations? This tension emphasizes the need to view research not as a neutral process, but as a dynamic interaction in which ethical and normative considerations play a central role.

To make participation effective and inclusive, it is essential to develop strategies that do justice to these complex local contexts. This requires not only attention to methodological choices such as low-threshold and accessible forms of participation, but also critical reflection on the power dynamics within participatory processes. Ensuring inclusivity requires a structural approach, in which the involvement of diverse groups of residents does not depend on random initiatives, but is embedded in sustainable policy.

## Methods and approach

### Case study approach

The 3 days inside case study was conducted using the PAR methodology, a research methodology that positions itself at the intersection of scientific knowledge development and practical improvement. The researchers were looking for an accessible and engaging way to connect with residents and gain insight into their lives. They found inspiration for this in Sanne Visser's doctoral research (24), in which she stayed in a community for an extended period of time to conduct research. This gave the researchers the idea of temporarily immersing themselves in the context by “living there” for 3 days. With pure intentions, they attempted to let go of the classic research frameworks and participate fully in the practice as researchers. This was based on the conviction that you can only see reality if you “enter” the system you are actually working in (17).

### Preparation

Prior to this 3-day stay, contact was made with local professionals to gain insight into ongoing initiatives that the research group could join, with the aim of helping and assisting where necessary. The entry point via the Professional Doctorate candidate in the context was essential in order to give this experiment a chance of being carried out within a reasonable time frame. Based on this inventory among the professionals, a program was put together and the professionals involved were informed about the presence of the researchers. Contact with residents was not based on predefined sampling criteria. Instead, researchers entered the community through the local welfare organization and asked where they could be of help or contribute to ongoing activities. As such, the “participants” in this study were not a fixed or pre-selected group, but consisted of residents who were present in and engaged with these activities during the 3-day period. A suitable place to stay for the research group was also sought. The preference was to stay in the middle of the community, but the options were limited. Due to the size of the group, the only hotel in the city was chosen.

In addition, consideration was given in advance to how the findings would be recorded during the 3 days. Logbooks were chosen over audio recordings or transcripts to allow researchers to remain fully present in the interactions and to prioritize relational engagement over data extraction. This approach aligns with the principles of PAR, in which building trust, reciprocity, and responsiveness to the context are central. Writing in logbooks enabled immediate reflection on experiences while minimizing disruption to natural flow of everyday interactions. This data was approached from three perspectives, after which the researchers reflected on their own actions – a process known as reflection on action. This will be explained in more detail further into this chapter.

### Implementation

Table 1 shows where the researchers were located and what working method they used at each location during the 3 days. What is not visible in the table, but was essential, is that the researchers were constantly engaged in a process of context exploration and relationship building, something that was not planned as a separate activity, but arose naturally through their presence.

**Table 1:** Overview working methods.

Location	Working method
Day 1	
Appingedam city center	Transect walk
Restaurant Paviljoen Overdiep	Fly on the wall
Hotel Het Wapen van Leiden	Go-along interviews
Neighborhood Westerkade	Transact walk
Neighborhood Opwierde	Transact walk
Day 2	
Hotel Het Wapen van Leiden	Go along interviews
City Garden De Eendracht	Go-along interviews
	Participant shadowing
Lunch Community Center Cadanz Welfare	Dialogical interview
City Garden De Eendracht	Go-along interviews
	Participant shadowing
Supermarket Albert Heijn	Fly on the wall
Coffee bar Stroopwoafeltje	Fly on the wall
Snackbar De Vesting	Fly on the wall
Cafe De Doofpot	Go-along interviews
Day 3	
Hotel Het Wapen van Leiden	Go-along interviews
Market Appingedam city center	Go-along interviews
	Participant shadowing

A mix of PAR methods was chosen, which are described below:

*Transact walk* is a systematic walk through an area together with residents or stakeholders. The researchers walked through Appingedam with residents and asked them to point out places that are important, problematic or meaningful (25).

*Fly on the wall* is observing without actively participating. The researchers watched and listened but interfered as little as possible (26).

*Go-along interviews* are interviews that take place while you are going somewhere or doing something together. The context is included in the conversation. While residents were going about their daily routine (e.g., the hotel employee or resident who was active in the city garden), the researchers asked questions (27).

*Participant shadowing* is “walking along” with a participant during their daily activities, as the researchers did when working in the city garden. They observed how the person acts and communicates (28).

*Dialogical interview* is a conversation in which the researcher and resident, in this case, construct meaning together. It is more about exchange than asking questions and getting answers. At the lunch in the welfare center, both parties (researchers and residents) contributed their experiences, asked each other questions and reflected together (29).

### Reflection on action

The observations and experiences of the research experiment were recorded in individual logbooks and organized according to the three perspectives. The logbooks were then discussed and analyzed with all those involved. Reflection on Action refers to the systematic review of one's own actions after a practical situation has ended. By taking a step back and reflecting analytically on what has happened, underlying assumptions, moments of choice and effects become visible. This reflection enables those involved to give meaning to their experiences and to critically examine the extent to which their actions were effective, appropriate or desirable. In the context of PAR, this form of reflection contributes to the collective formation of knowledge, because it invites researchers to make their practices explicit, discuss them and adjust them where necessary. An essential feature of PAR is the cyclical nature of the research process. Usually, successive phases of planning, acting, observing, and reflecting are used. Reflection plays a central role within this cycle, because it forms the bridge between experience and insight, thereby laying the foundation for subsequent actions. In this context, the Reflection on Action method, as described by Schön (31), is highly relevant.

### Three perspectives

The three perspectives are intended to clarify the research process: (1) the individual (I), how the researcher experiences and feels it; (2) the collective (we), what feelings it evokes in others; and (3) the systemic level (the system). This approach is inspired by the work of Reason and Torbert (30), who distinguish between first-person, second-person, and third-person action research as complementary forms of reflective practice. The three perspectives are used as a tool to organize observations and experiences.

## First-person action research: the “I” level

The first perspective concerns first-person action research, in which the researcher systematically reflects on their own actions, with explicit attention to intentions, behavioral strategies and the consequences of those actions (30). At this level, the researcher examines the ethical dilemmas that arise in practice, attempts to understand underlying value tensions and the associated emotions within themselves, and adapts their actions based on these insights. This cyclical reflection enables the researcher to develop moral competencies and generate knowledge in and through Reflection on Action (30). In the case of 3 days inside, the researchers took notes every day and collected them in an individual logbook.

### Second-person action research: the “we” level

In the second perspective, research takes place in dialogue with others, within a community of researchers and stakeholders. In 3 days inside, this involved the residents, professionals and researchers. This second-person action research focuses on joint reflection and meaning-making, in which experiences, feelings, and ideas are shared (30). In the case of 3 days inside, the researchers discussed specific issues with others, actively listened to different perspectives, and, where necessary, involved other stakeholders in the reflection process. Every day, joint reflection took place in informal settings—such as during conversations around picnic tables or walks—as well as in more structured reflective dialogues, such as during breakfast and dinner. This joint reflection leads to new insights and concrete actions, which in turn are again the subject of reflection in the next cycle.

### Third-person action research: the system level

Finally, third-person action research also takes place, enabling systemic learning within broader communities consisting of actors who do not necessarily meet each other. This form of research facilitates shared critical reflection in broader contexts, such as scientific publications, networks, and professional learning communities (30).

Taken together, these perspectives suggest that meaningful lessons learned and practical implications at the system level depend on insights generated the “I” and “We” levels of reflection and collective meaning-making.

## Lessons learned and practical implications

The analysis of 3 days inside shows that PAR is not only a methodological choice, but a continuous process of relational positioning, ethical alignment and learning to deal with unpredictability. The practical experiences and logbook reflections (referred to as R1—R5 in the text) offer the researchers a series of lessons that are important for future research and policy practices. These lessons learned should not be understood as direct outcomes of specific situations or methods; rather, they emerge from recurring patterns across experiences and reflections, making them more generically applicable to PAR practice. Table 2 provides an overview of these lessons learned formulated through the “I” and “We” level of reflection, which are further elaborated below, followed by a translation into practical implications framed through the reflection on system level.

**Table 2:** Overview lessons learned and practical implications.

Key insights	Lessons learned	Practical implications
Role of the researcher	Researchers are never neutral; their presence, vulnerability and positioning shape the process.	Embrace reflexivity, acknowledge influence, and prepare researchers to work with doubt and shifting roles.
Relational **vs**. transactional working	Symbolic or instrumental participation creates mistrust; relational presence builds trust but is fragile.	Invest in long-term relationships, continuity, and transparency about research interests to avoid tokenism.
Ethical positioning	Ethics emerge in encounters; informed consent is relational, not a static document.	Treat ethics as ongoing dialogue; adapt procedures flexibly to local sensitivities.
Participatory observation	Everyday activities and informal encounters yield richer insights than structured interviews.	Embed researchers in community life; allocate time/resources for shared activities as key methodology.
System world vs. living world	Policy intentions often clash with lived reality; inequalities are visible on the ground.	Ensure policymakers/researchers are physically present in neighborhoods to design responsive and fairer policies.
Trust and long-term presence	Trust grows through consistency, reciprocity, and everyday closeness, not short projects.	Build structural, long-term commitments and embed participatory research in existing networks and routines.

### Role of the researcher

#### Lesson learned

The logbooks of all researchers show that the role of the researcher was constantly under discussion. As R4 stated: “What does it mean when control is released?” Letting go of control and giving space to co-creation proved essential, but at the same time confrontational. It was precisely in those moments of uncertainty that new forms of collaboration could emerge, if researchers were willing to acknowledge their own vulnerability and accept that their presence always constitutes an intervention in itself.

These reflections were accompanied by questions about legitimacy and positioning. As R2 noted: “Who am I to intervene here? Maybe the people themselves don't see it as a problem at all.” At the same time, there was a tension between authenticity and connection: “I contribute from my own identity. At the same time, I have a tendency to assimilate. Otherwise, acceptance comes under pressure.”

In addition to doubt and self-examination, there was also a sense of responsibility:

*I feel responsible for the idea, the process, and the outcome. I hope everyone thinks it's a good idea*. (R3)

These quotes make it clear that researchers are not merely neutral observers, but become part of the dynamics, tensions, and vulnerabilities of the research process themselves.

### Practical implications

PAR requires reflexivity and the courage to acknowledge one's own position and influence. The researcher's attitude is crucial for fostering participation, and, in this case, reciprocity. Attitude is something that must be practiced, and this case study demonstrates that an immersive experiment like this can be used to develop and improve it. By spending time in the community with fellow researchers, engaging with residents without a predetermined agenda, focusing on reciprocity, and reflecting on these interactions, researchers can actively cultivate the attitudes needed for meaningful participatory research. Researchers should not hide behind a neutral role but explicitly use their presence as a relational contribution to the co-creative process. Support structures and training can help researchers embrace uncertainty, vulnerability, and responsibility as integral parts of the research.

### Relational vs. transactional working

#### Lesson learned

This experience directly touches on the tension between relational and transactional working. During a conversation with residents in the city garden, it was striking how sensitive they are to symbolic participation. One resident said:

*She never came here… but at the opening she was at the front!* (R1)

It made it clear that transactional approaches that use residents instrumentally for policy goals or public visibility arouse mistrust.

Relational working, on the other hand, requires a sustained presence, recognition of agency and investment in trust. A single appearance at a festive moment is not enough; it is about the long-term commitment of constantly returning, participating, and listening. On the other hand, relational practices were certainly appreciated. For example, one resident said of an area director:

*She gave us confidence; we feel she listens to us*. (R1)

Nevertheless, it also became apparent in the city garden volunteer group that relational intentions were sometimes interrupted by transactional patterns. R4 wrote: “Strong hierarchy among the volunteers. You would expect a relational bond, but what you find is a transactional setting.”

Although the researchers initially intended to approach the experiment on a relational basis, an underlying paradox soon became apparent. The starting point was reciprocal contact and equal encounters, but at the same time, they were motivated by the desire to obtain answers to their own research questions. This tension was aptly expressed by one of the researchers with the concept of “change”: the idea that by offering support or being present now, the opportunity would arise later to put one's own question back to the community.

#### Practical implications

To avoid symbolic or transactional participation, researchers, and professionals need to prioritize continuity, presence, and reciprocity. Relational working requires sustained commitment and transparency about research motives. Policies should foster conditions that support long-term engagement rather than one-off appearances or instrumental uses of resident input.

### Ethical positioning

#### Lesson learned

This leads to the following lesson: ethical positioning is not just a matter of pre-established protocols, but a continuous process in interaction with residents. During an evaluation moment in the local coffee bar, R2 wrote: “They wondered why we were all sitting there with notebooks.” This showed that taking notes, which is natural for researchers, can be perceived by residents as distant or controlling. It became apparent that informed consent is not a static document but takes time to shape in dialogue and explanation.

A recurring reflection concerned the ethical positioning of the researchers themselves. As one noted:

*It doesn't feel right not to share who we are and what we are here to do. You can't observe if the person doesn't know anything about it*. (R3)

At the same time, the researchers struggled with the formality of ethical procedures:

*The complicated thing about asking for informed consent is that it should actually be very natural. Because once people sign, it's set in stone and it's no longer their own story*. (R4)

#### Practical implications

These reflections emphasize that ethics must be treated as a relational and ongoing process. Researchers should engage in transparent dialogue and adapt to how consent and trust are shaped in everyday encounters. Institutions and ethics committees should recognize that rigid, one-off procedures may hinder genuine engagement and should support flexible, context-sensitive approaches.

### Participatory observation

#### Lesson learned

The value of the research lay not in planned interviews or predetermined questions, but in small, everyday encounters. For example, during a few hours of working together in the city garden, informal conversations arose that deepened during a joint lunch in the welfare center. In that informal setting, one resident said:

*We are all on the same level. I hate judging people*. (R5)

Actual participation in activities proved to be a powerful catalyst. As R3 noted: “When you come to contribute something (come to help), how easily conversations get started without even consciously bringing up topics.” It also became clear how physical effort contributes to trust:

*Participating and working hard shows that you are investing in someone else, and that makes them open up to you*. (R1)

R2 put it aptly: “An afternoon spent observing in an everyday setting is richer than an interview.”

#### Practical implications

Participatory observation through proximity, involvement, and active immersion is indispensable for context-sensitive insights. Researchers should plan for informal, everyday engagement as part of their methodology. Funding and policy structures need to value and allow time for this kind of embodied presence, recognizing its contribution to richer, more grounded knowledge.

### System world and living world

#### Lesson learned

The tension between the system world and the living world became apparent on several occasions. While working in the city garden, a resident said:

*They really don't realize how much work it is from behind their desks*. (R3)

This highlighted the gap between policy intentions and residents lived realities.

R5 described during the neighborhood tour: “Although I was familiar with the issues, it only really sank in when we walked through the neighborhoods.” R4 noted the symbolism of inequality in the built environment: “Some houses are completely new, while others on the same street are dilapidated… The division seems huge. Question marks seen in Westerdraai: ‘neighborhood-worthy, equal.”'

#### Practical implications

Bridging the gap between policy systems and lived experience requires policymakers and researchers to be physically present in the neighborhood. This allows them to perceive inequalities and respond in context-sensitive ways. Policies should include mechanisms for regular, direct engagement in everyday settings rather than relying only on documents and meetings.

### Trust and long-term presence

#### Lesson learned

The experiences emphasize the importance of trust and long-term presence. One resident noted:

*This experimental space is made possible in part… by long-term presence*. (R4)

Trust does not arise from *ad hoc* projects or one-off interventions, but grows through consistent presence and shared time, both in formal and informal settings.

The logs show how trust cannot be enforced but grows gradually. During the final visit to the market, one researcher observed:

*We did not ask anyone to participate in our research. Yet, after 3 days, the threshold for participating in projects seems to have been lowered*. (R5)

Active participation laid the foundation for trust: “Participating, immersing yourself, giving something back works for trust and building relationships with people.” Or R1 summarized: “Bring something to the table, instead of just being the qualitative interviewer.”

#### Practical implications

Trust requires continuity and reciprocity. Researchers and institutions need to invest structurally in long-term presence and embed participatory research within existing networks, although it is a time- and labor-intensive process. Only consistent and reciprocal engagement can build durable trust and collaboration.

### Research as a dynamic process

The 3 days inside case study makes clear that PAR is best understood as a dynamic and relational process rather than a linear or neutral method. Research always embodies normative choices tied to values, positions, and context. As R2 summarized: “Research is never neutral…” The logbook reflections show how ethical and methodological decisions cannot be fixed in advance, but continually arise through encounters and dialogue.

The locations and settings in which the research unfolded played a crucial role in shaping the process. Everyday spaces, such as a pub, proved to be meaningful sites for knowledge exchange:

*It is not always necessary to actively approach people. A pub is an excellent opportunity to engage in conversation with different generations*. (R3)

Such observations highlight that valuable insights often emerge in informal, everyday contexts rather than in formal research settings.

At the same time, the research revealed broader social tensions in the community, where hope and resignation coexist side by side. As R4 noted: “A stark difference in belief in society. From day in, day out, rolling up your sleeves… to systematic resignation: ‘It is what it is.”' This underlines how PAR is always embedded in complex emotional and social dynamics.

Overall, the case study shows that PAR requires flexibility, openness, and critical self-reflection. Researchers must continuously reposition themselves, adapt to context, and acknowledge their own influence. Only by embracing unpredictability and working in close proximity to everyday life can research do justice to the complexity of the living environment in which it takes place.

## Discussion

The 3 days inside case study shows that research in Appingedam is not a separate activity but is integrally interwoven with practice. The situations and quotations described make it clear that proximity, relational investment and long-term presence form the basis for meaningful collaboration.

**Figure 1:**
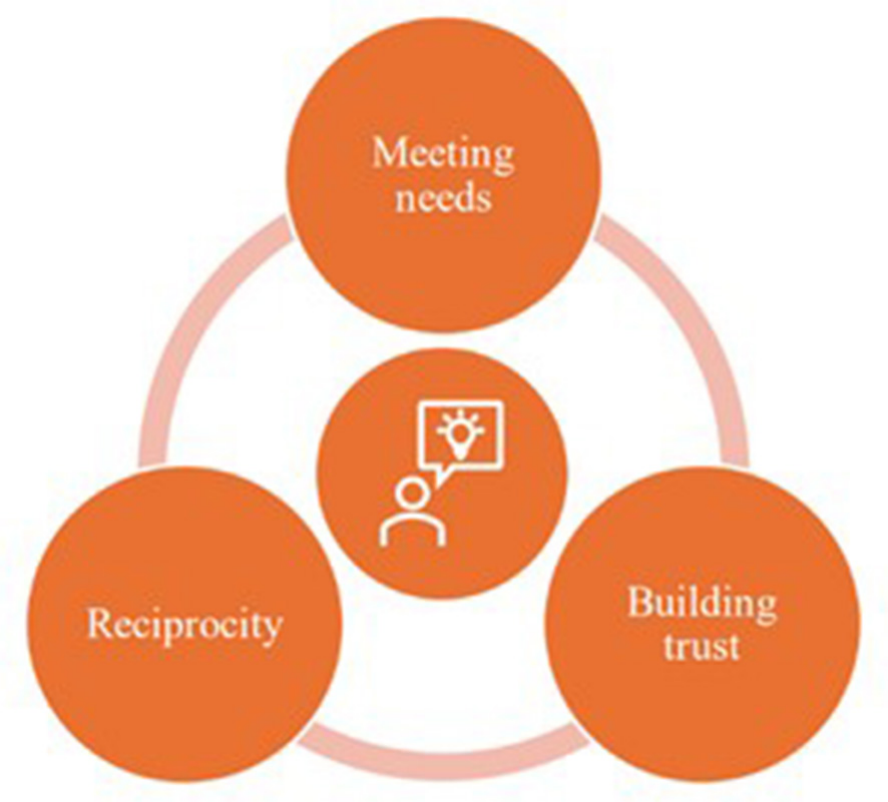
Cyclical model.

Figure 1 illustrates this dynamic as a cyclical model: when needs are met, trust can be built, which in turn creates the conditions for reciprocity. Reciprocity again generates new opportunities to meet needs, so that the cycle continues and relationships deepen over time. The researcher is placed at the center of this cycle, not as a controlling actor but as a relational presence. It is not only the professional role that matters here, but also the person behind the researcher: their personality, values, vulnerabilities and way of engaging with others shape how the cycle unfolds. This perspective makes visible that the researcher is never outside of the process, but always part of the dynamics they seek to understand.

At the same time, the 3 days inside case study yielded methodological and personal insights and continues to yield benefits for the local community. It revealed the tensions that arise within PAR, such as balancing reciprocity and one's own research interests. For the researchers, this was also a learning experience: by stepping over the threshold into practice in order to perceive the complexity of the context more clearly and to rethink the role of the researcher in relation to fostering reciprocity. For the community, small but tangible benefits were created, such as support in preparing the city garden for winter and opportunities to connect informally with researchers and professionals. These encounters may contribute to building trust and opening space for more constructive dialogue with institutions in the future. This community case study thus serves as an awareness-raising document, useful for both research and policy. The lessons learned transcend the local context and offer a mirror for anyone working on participation and health in vulnerable regions. It is an invitation to no longer view research and policy as linear processes, but as relational processes in which reciprocity, proximity, and trust are central.

### Limitations

A limitation of this study is that we could only question a limited diversity of residents, locations, and activities. This may have influenced the range of perspectives and experiences that were captured. To strengthen general practical utility of this approach, we recommend future studies to examine its applicability in other contexts.

Another point to consider is that the researchers' own values, experiences, and expectations likely shaped the interactions and interpretations. While this reflexivity is inherent to PAR, it also underlines the importance of being transparent about the positionality of the researchers.

### Future research

These experiences raise questions for further exploration. At the individual level: how can the researcher cultivate the tacit knowledge—such as reflexivity, openness, and reciprocity needed to engage meaningfully in participatory research? At the research level: how do the chosen methods support or constrain reciprocity within existing participatory research structures, for example when facing ethical dilemmas or questions of informed consent? And at the system level: what conditions are necessary to embed participatory research sustainably in policy and practice structures, including providing space for the researcher to engage meaningfully with the community in context?

## Data Availability

The original contributions presented in the study are included in the article/supplementary material, further inquiries can be directed to the corresponding authors.
